# Evaluation of a two step testing algorithm to improve diagnostic accuracy and stewardship of *Clostridioides difficile* infections

**DOI:** 10.1186/s13104-023-06398-9

**Published:** 2023-08-14

**Authors:** Caitlin C. Bettger, Stephanie E. Giancola, Robert J. Cybulski, Jason F. Okulicz, Alice E. Barsoumian

**Affiliations:** 1 Department of Internal Medicine, Brooke Army Medical Center, JBSA Fort Sam Houston, TX USA; 2 Department of Pharmacy, Brooke Army Medical Center, JBSA Fort Sam Houston, TX USA; 3 Department of Pathology and Laboratory Services, Brooke Army Medical Center, JBSA Fort Sam Houston, TX USA; 4 Infectious Disease Service, Brooke Army Medical Center, JBSA Fort Sam Houston, TX USA

**Keywords:** *Clostridioides difficile* infection, Diagnostic stewardship, Two-step testing

## Abstract

In response to national guidelines, we implemented a two-step testing algorithm for *Clostridioides difficile* in an effort to improve diagnostic accuracy. Following implementation, we analyzed treatment frequency between discordant and concordant patients. We found that the majority of discordant cases were treated with no significant differences in patient characteristics or outcomes between the concordant and discordant groups. Additionally, there were no differences in outcomes when discordant patients were further stratified by treatment status. Given little added diagnostic accuracy with the addition of EIA toxin testing, our facility resumed diagnosis by PCR testing alone. Further studies are needed to investigate alternative processes for improvement in diagnostic accuracy aside from toxin EIA testing including stool submission criteria and educational programs.

**Supplementary Information:**

The online version contains supplementary material available at 10.1186/s13104-023-06398-9.

## Introduction

Although nosocomial *Clostridioides difficile* infections (CDIs) are decreasing nationwide, [1] it continues to be a major threat leading to 12,800 deaths per year [2] and contributes billions of dollars in healthcare costs annually [1, 3]. CDI is a clinical diagnosis prompted by patient presentation and informed by laboratory testing. However the diagnosis is complicated by the nature of the information made available through laboratory assays [4]. Toxigenic cell culture assays and cell culture cytotoxicity neutralization assays are generally considered gold standards for the detection of toxigenic *C. difficile* [5], but are not generally feasible in a clinical laboratory setting. Enzyme immunoassays (EIA) testing for the presence of toxin have suboptimal analytical sensitivities and can lead to missed cases of clinically important CDI [6, 7]. Newer nucleic acid amplification based assays, while showing improvement in case detection, have been shown to have poor clinical specificity [6–8]. Best practices for laboratory testing in support of CDI diagnosis remains a subject of debate [9]. In response, the Infectious Disease Society of America (IDSA) published new recommendations in 2018 for laboratory testing of CDI. If possible, the institutions are to restrict CDI testing only for those patients who meet strictly defined clinical criteria (those with unexplained, acute onset of ≥ 3 unformed stool in 24 h, who are not also receiving laxatives) [4]. Otherwise, institutions may implement a two-step testing algorithm, where a PCR based assay is performed first, and followed by a second assay such as the toxin EIA, to assist in the diagnosis of CDI.

A two-step testing algorithm can present a diagnostic conundrum. PCR positive and EIA toxin negative (PCR+/EIA-), also referred to as discordant results, could mean that *C. difficile* is present in the sample but not causing clinical disease, such as representing colonization or previous infection [10, 11]. However, given suboptimal sensitivity of EIA, some discordant samples will likely represent true CDI and may lead to missed diagnoses. Indeed, several studies found no difference in clinical outcomes between discordant and concordant patients, irrespective of treatment [11, 13, 14]. Accurate distinction between colonization and CDI is desirable because it results in cost savings, appropriate antimicrobial stewardship, accurate diagnosis, and decreases risks for vancomycin resistant *Enterococcus* colonization [12].

Our institution relied on a PCR-only testing strategy at the time of the 2018 guideline publication. Upon publication, the antimicrobial stewardship, infection control, and microbiology teams reviewed practices. Our institution was unable to implement stool submission criteria at the time because our laboratory techs were unable to view the same electronic medical record as providers (where laxative and stool count would have been documented). Based on our facility’s hurdles to enforcing stool submission criteria, we sought to implement a two-step testing algorithm for CDI, and carefully evaluate outcomes given the potential limitations of this strategy.

## Methods and materials

Our facility, a tertiary care center, adopted a two-step testing strategy on 16 August 2018. Initially, EIA results were delayed by approximately 40–60 min, however, this was remedied within the first month of implementation. Subsequent EIA results were released at the same time as PCR results. Prior to implementation, this change and education was administered through departmental leadership. Additional information about discordant tests was populated in the laboratory results interpretation section of CDI testing labs in our electronic health record. Accessing this guidance required providers to navigate to a separate tab in the results screen. In the first month post-implementation, providers were contacted with supplemental information about discordant testing via encrypted e-mail (for outpatients) or via an antimicrobial stewardship note (for all inpatients).

All CDI inpatient and outpatient testing results from 17 August 2018–30 September 2019 were retrieved from the electronic medical record. Xpert *C. difficile* (Cepheid, Sunnyvale, CA, USA) assay was used for all PCR testing. Wampole *C. difficile* Testing, Tox A/B II EIA 96-T (Grayline Medical, Norwalk, CA, USA) was used for all toxin EIA testing. Only PCR + tests with subsequent EIA toxin data were included in this analysis. Remaining data was collected through inpatient and outpatient chart review. Demographic data including gender, age, and inpatient status were collected. Risk factors for CDI including history of previous CDI and antibiotic use within the prior 30 days were also recorded. Preceding laxative use and median daily stool count were annotated when available. Laboratory data, such as white blood cell count, albumin, and creatinine on the day of the positive test were also obtained. Patients were recorded as having severe CDI if they had WBC > 15,000 cells/mL and/or serum creatinine ≥ 1.5 mg/dL, or if they had fulminant colitis [3]. Clinical features including treatment decision, antibiotic choice, sub-specialty consultation (Infectious Disease and/or Gastroenterology), length of stay, recurrent CDI within 30 days, readmission with CDI within 30 days, and CDI-related mortality at 30 days were also retrieved from chart review.

Concordant and discordant groups were compared by Chi square (categorical variables) and Mann-Whitney-U (continuous variable). Characteristics and outcomes of treated cases based on discordant status were compared. Subgroup analysis was performed on discordant cases compared by treatment status to evaluate for identifiable factors associated with treatment decision. Chi Square was again used for categorical variables and Mann-Whitney-U for continuous variables in this sub-analysis.

## Results

A total of 216 PCR + tests from 215 patients were recorded during the study time period. Of these, 155 (71.8%) were discordant. Demographics, laboratory data, and risk factors for CDI were similar between groups (Table 1; p > 0.05 for gender and age). Compared to discordant cases, concordant cases were more frequently hospitalized (59% vs. 43.9%; p = 0.05), had a higher median daily stool count (5 [4–7] vs. 4 [2–6], p = 0.03), met criteria for severe CDI (33.3% vs. 18.7%; p = 0.05), received treatment (95.1% vs. 66.5%; p < 0.01) and were readmitted in 30 days with CDI (8.3% vs. 1.3%; p = 0.02). Importantly, mortality did not differ between the groups. There was no significant difference in laboratory data including median white blood cell count and medium serum creatinine between concordant and discordant groups. Additionally, CDI risk factors (antibiotic use within the last 30 days and history of CDI) were similar.

**Table 1:** Characteristics and outcomes of patients with concordant and discordant tests

Characteristics	All (n = 216)	PCR+/EIA+ (n = 61)	PCR+/EIA- (n = 155)	P-Value
*Demographic Information*				
Gender, male	106 (49.1%)	27 (44.3%)	79 (51%)	0.49
Median age (years)	56 (30, 72)	60 (43–75)	55 (27–71)	0.08
*Laboratory Data*				
Median WBC (10^3^ cells/mL) (n = 139)	9.8 (6.1–13.4)	11.3 (6.74–15.6) (n = 48)	8.66 (5.76–12.5) (n = 91)	0.13
Median serum creatinine (mg/dL) (n = 141)	0.87 (0.66–1.19)	0.85 (0.67-1.0) (n = 50)	0.89 (0.66–1.25) (n = 91)	0.29
*Risk factors for CDI*				
Antibiotic use within 30 days (n = 203)	107 (52.7%)	36 (60.0%) (n = 60)	71 (49.7%) (n = 143)	0.18
History of CDI	27 (12.5%)	8 (13.1%)	19 (12.3%)	0.86
*Clinical Features*				
Laxative use (n = 205)	29 (15.6%)	6 (10.3%) (n = 58)	23 (15.6%) (n = 147)	0.33
Median daily stool count (n = 147)	4 (2.5-6)	5 (4–7) (n = 42)	4 (2–6) (n = 105)	0.03
Hospitalized	104 (48.1%)	36 (59.0%)	68 (43.9%)	0.05
Severe CDI (n = 139)*	33 (23.7%)	16 (33.3%) (n = 48)	17 (18.7%) (n = 91)	0.05
*Outcomes*				
Treated for CDI	161 (74.5%)	58 (95.1%)	103 (66.5%)	< 0.01
Median length of stay (days) (n = 104)	8 (4–16)	6 (4–11) (n = 36)	9 (3.75–18.3) (n = 68)	0.3
Mortality at 30 days (n = 200)	9 (4.5%)	3 (5.3%) (n = 57)	6 (4.2%) (n = 143)	0.74
Readmission for CDI at 30 days (n = 214)	7 (3.3%)	5 (8.3%) (n = 60)	2 (1.3%) (n = 154)	0.02

All data expressed as number (%) or median (IQR)

*White blood cell count > 15,000 cells/mL and/or serum creatinine ≥ 1.5 mg/dL

A subgroup analysis compared treated and untreated discordant patients (Table 2). There was no difference in gender, median age (56 [27.5–71] vs. 51 [26-69.5] years, p = 0.71), hospitalization (44.7% vs. 42.3%, p = 0.78), history of CDI (11.7% vs. 15.7%, p = 0.60), antibiotic use within 30 days (48.9% vs. 51.0%, p = 0.81), severe CDI (23.3% vs. 9.7%, p = 0.16), readmission (2.0% vs. 0%, p = 0.55), or mortality (3.2% vs. 6.1%, p = 0.67) among groups. Additionally, clinical indicators of patient acuity (i.e. laboratory data) were not significantly different between treated and untreated discordant cases. However, treated discordant cases had a higher median daily stool count (4 [3–7] vs. 3 [1–5], p = 0.02). A high proportion of discordant cases evaluated by Infectious Disease (73.9%, n = 23) or Gastroenterology (61%, n = 54) received treatment.

**Table 2:** Characteristics and outcomes of treated and untreated patients with discordant tests

Characteristics	All (n = 155)	Treated (n = 103)	Untreated (n = 52)	P-Value
*Demographic Information*				
Gender, male	79 (51.0%)	50 (48.5%)	29 (55.8%)	0.40
Median age (years)	55 (27–71)	56 (27.5–71)	51 (26-69.5)	0.71
*Laboratory Data*				
Median WBC (10^3^ cells/mL) (n = 91)	8.75 (5.76–12.5)	9.68 (6.32–13.2) (n = 62)	7.61 (5.76–11.1) (n = 29)	0.28
Median serum albumin (g/dL) (n = 76)	3.6 (3.1–4.1)	3.6 (3-4.1) (n = 45)	3.6 (3.25–4.1) (n = 31)	0.76
Median serum creatinine (mg/dL) (n = 91)	0.89 (0.65–1.29)	0.95 (0.68–1.42) (n = 59)	0.84 (0.62–1.06) (n = 32)	0.09
*Risk factors for CDI*				
Antibiotic use within 30 days (n = 143)	71 (49.7%)	45 (48.9%) (n = 92)	26 (51.0%) (n = 51)	0.81
History of CDI (n = 154)	20 (13.0%)	12 (11.7%) (n = 103)	8 (15.7%) (n = 51)	0.60
*Clinical Features*				
Laxative use (n = 147)	23 (15.6%)	16 (16.2%) (n = 48)	7 (14.6%) (n = 99)	0.81
Median daily stool count (n = 105)	4 (2–6)	4 (3–7) (n = 68)	3 (1–5) (n = 37)	0.02
Severe CDI* (n = 91)	17 (18.7%)	14 (23.3%) (n = 60)	3 (9.7%) (n = 31)	0.16
Gastroenterology consulted	54 (34.8%)	33 (32.0%)	21 (40.4%)	0.30
Infectious disease consulted	23 (14.8%)	17 (16.5%)	6 (11.5%)	0.48
*Outcomes*				
Median length of stay (days) (n = 69)	9 (3.5–18.5)	10 (4.5–25) (n = 47)	6 (3.5–12.5) (n = 22)	0.23
Mortality at 30 days (n = 143)	6 (4.2%)	3 (3.2%)(n = 94)	3 (6.1%) (n = 49)	0.67
Readmission for CDI at 30 days (n = 154)	2 (1.3%)	2 (2.0%) (n = 102)	0 (0%) (n = 52)	0.55

All data expressed as number (%) or median (IQR)

*White blood cell count > 15,000 cells/mL and/or serum creatinine ≥ 1.5 mg/dL

Antibiotic treatment regimens were reviewed for all treated cases. The majority of all cases were treated with guideline-recommended regimens (87.4%) regardless of discordant status. Oral vancomycin was the most commonly prescribed antibiotic (65.2%), followed by fidaxomicin (14.9%), metronidazole (10.6%), and combination therapy (9.3%). Characteristics and outcomes were similar amongst treated discordant and concordant cases (Supplemental Table 1)

## Discussion

The laboratory diagnosis of CDI remains hotly debated; however, both the IDSA and European Society of Clinical Microbiology and Infectious Disease (ESCMID) recommend multi-step algorithms in order to improve the diagnostic accuracy of CDI [4, 17]. Prior surveys have suggested that laboratory diagnosis of C difficile infections vary widely. ESCMID currently recommended algorithms include some combination of glutamate dehydrogenase (GDH) EIA, toxin EIA, or PCR followed by a confirmatory test (either EIA detecting GDH or toxin or PCR depending on what was used in the first round). IDSA recommended algorithms include EIA for GDH plus toxin EIA, EIA for GDH plus toxin EIA arbitrated by PCR, PCR plus toxin EIA, or PCR alone with pre-agreed institutional criteria for patient stool submission.

Implementation of a two-step testing strategy for the diagnosis of CDI at our institution led to withholding antibiotic treatment in approximately 34% of discordant samples. We did not identify clinically significant differences or outcomes among patients with concordant or discordant samples. Additionally, there were no identifiable differences in patient characteristics or adverse outcomes including recurrent CDI, readmission, or mortality between treated and untreated discordant patients.

Our primary aim in revising the testing strategy at our institution was to improve the diagnosis of clinically significant CDI. While discordant laboratory results may have helped providers distinguish between colonization in the untreated minority of cases, the majority of discordant cases at our institution were deemed clinically significant and were treated. Our analysis failed to identify clinically significant differences in patient characteristics between treated and untreated discordant cases which may have explained decisions to treat. As such, it is likely that unmeasured factors guided the clinical decision making in these cases and allowed providers to diagnose CDI in the absence of concordant laboratory data. Interestingly, even expert consultation by Infectious Disease or Gastroenterology physicians led to a high proportion of treatment of discordant cases (61–74%). This argues against the hypothesis that these patients were treated based on lack of expertise with two-step testing or misattribution of colonizing isolates as clinically significant.

With the exception of CDI related readmission, there were no statistical difference in outcomes, most notably mortality, between discordant and concordant cases. Our findings are similar to previous studies [9, 16], which suggests that the identification of toxin positivity is less relevant in patients with clinically significant disease. Additionally, this further supports the recommendation for PCR testing alone when pre-test probability is improved with stool submission criteria [4].

Similar to previous studies [11, 13, 14], we observed no increase in adverse events when discordant cases were not treated. While this has been previously taken as support that discordant cases are most likely representative of colonization as opposed to true infection, the actual significance of these cases is unclear. It may be that a proportion or all of the treated discordant cases were accurately assessed as clinically significant and treated with satisfactory outcomes, or may just be underpowered to determine differences.

Diagnostic accuracy must be balanced against cost considerations associated with over treatment. In our study, two step testing resulted in a significant cost savings compared to PCR testing alone. Estimated drug costs based on current drug price of treatment with fidaxomicin, vancomycin, or metronidazole monotherapy over the analysis period was $54,868.65. Calculated drug costs if all 216 cases were treated (as they likely would be had diagnosis been made on PCR alone) with the same proportion of agents would be $73,311.80, resulting in an estimated drug cost savings of $18,443.15 over the 54 week period. It is unlikely that costs saved with elimination of toxin EIA would offset this savings.

Our findings highlight that the interpretation of laboratory tests for CDI remains complex [6]. Interestingly, a low effort intervention to reduce excess sample submission resulted in a reduction in false-positive nosocomial CDI diagnoses as well as a reduction in excess test ordering at an institution relying solely on a one-step testing strategy [15]. This team utilized a one-time educational intervention, distribution of information, and created an informational screen-saver with CDI diagnostic criteria as part of their effective campaign. This may suggest that some simple approaches, such as visual reminders, may be useful in decreasing inappropriate lab ordering, which would lead to gains in diagnostic stewardship. As such, this may be an opportunity to improve diagnostic stewardship for CDI even when institutions are unable to abide by enforcing stool sample submission criteria which are recommended by the IDSA [4]. Following this analysis, we refined our CDI diagnostic strategy to return to a one-step algorithm, with EIA performed by request, and are developing additional interventions to improve our testing approach. Our institution has now moved to a single electronic medical record, which may facilitate the implementation stool submission criteria in the future.

Limitations of this study were that this project was performed in a single-center with a relative small number of cases over the period studied which may be underpowered to identify small differences.

## Conclusions

We implemented and evaluated a two-step testing algorithm in response to national guidelines and in an attempt to improve diagnosis of CDI. However, the algorithm did not change clinical management as the majority of discordant cases were treated as true infection. Further studies are needed to address the improvement of diagnostic accuracy with the implementation of other measures including stool submission criteria and provider education programs.

### Electronic supplementary material

Below is the link to the electronic supplementary material.

Supplementary Material 1

## Data Availability

All data generated or analyzed during this study are included in this published article.

## Abbreviations

CDI: *Clostridioides difficile* infection
EIA: enzyme immunoassay
PCR: polymerase chain reaction
WBC: white blood cell count
PCR+/EIA+: concordant tests
PCR+/EIA-: discordant tests
