# Supplementary material for: Evaluation of a two step testing algorithm to improve diagnostic accuracy and stewardship of Clostridioides difficile infections
Source: BMC Res Notes. 2023 Aug 14;16:172. doi: 10.1186/s13104-023-06398-9 (PMC10426052; doi:10.1186/s13104-023-06398-9)
Supplement: Supplementary file 1 — Supplementary Material 1 [file 13104_2023_6398_MOESM1_ESM.docx]

| Characteristics | All  (N=161) | Treated Discordant Tests (N=103) | Treated Concordant Tests  (N=58) | P-Value |
| --- | --- | --- | --- | --- |
| *Demographic Information* |  |  |  |  |
| Male Gender | 76 (47.2%) | 50 (48.5%) | 26 (44.8%) | 0.65 |
| Age | 59 (31.5, 72) | 56 (27.5, 71) | 59.5 (43.5, 75) | 0.14 |
| *Laboratory Data* |  |  |  |  |
| WBC (10^3^ cells/mL)  (N=107) | 10.2 (6.5, 14.0) | 9.68 (6.32, 13.2) | 11.3 (6.8, 15.5) | 0.30 |
| Albumin (g/dL)  (N=86) | 3.5 (3.0, 4.1) | 3.6 (3, 4.1) | 3.5 (3, 4.1) | 0.70 |
| Creatinine (mg/dL)  (N=106) | 0.87 (0.67, 1.33) | 0.95 (0.68, 1.42) | 0.84 (0.66, 0.96) | 0.14 |
| *Clinical Features* |  |  |  |  |
| Inpatient | 81 (50.3%) | 46 (44.7%) | 35 (60.3%) | 0.06 |
| First episode | 142 (88.2%) | 91 (88.3%) | 51 (87.9%) | 0.94 |
| Recurrence | 19 (11.8%) | 12 (11.7%) | 7 (12.1%) |  |
| Severe (N=105) | 29 (27.6%) | 14 (23.3%) | 15 (33.3%) | 0.26 |
| *Consultation and Treatment* |  |  |  |  |
| ID Consult | 28 (17.4%) | 17 (16.5%) | 11 (19.0%) | 0.69 |
| GI Consult | 46 (28.6%) | 33 (32.0%) | 13 (22.4%) | 0.19 |
| Treated correctly (N=159) | 139 (87.4%) | 87 (84.5%) | 52 (92.9%) | 0.13 |
| Fidaxomicin | 24 (14.9%) | 9 (15.5%) | 15 (14.6%) | 0.22 |
| Vancomycin | 105 (65.2%) | 67 (65.0%) | 38 (65.5%) | 0.22 |
| Metronidazole | 17 (10.6%) | 14 (13.6%) | 3 (5.2%) | 0.22 |
| Treated with multiple agents | 15 (9.3%) | 7 (6.8%) | 8 (13.8%) | 0.22 |
| *Outcomes* |  |  |  |  |
| 30 day mortality (N=148) | 5 (3.4%) | 3 (3.2%) | 2 (3.7%) | 0.87 |
| Recurrent C Diff (N=159) | 26 (16.4%) | 15 (14.9%) | 11 (19.0) | 0.50 |
| Readmission (N=159) | 7 (4.4%) | 2 (2.0%) | 5 (8.8%) | 0.45 |

Supplementary Table S1: Characteristics and outcomes of treated cases.

All data expressed as number (%) or median (IQR)
*White blood cell count >15,000 cells/mL and/or serum creatinine ≥1.5 mg/dL
